# L-type lectin receptor kinases: New forces in plant immunity

**DOI:** 10.1371/journal.ppat.1006433

**Published:** 2017-08-17

**Authors:** Yan Wang, Klaas Bouwmeester

**Affiliations:** 1 Laboratory of Phytopathology, Wageningen University, Wageningen, The Netherlands; 2 Department of Plant Pathology, Nanjing Agricultural University, Nanjing, China; 3 Plant-Microbe Interactions, Department of Biology, Utrecht University, Utrecht, The Netherlands; THE SAINSBURY LABORATORY, UNITED KINGDOM

## Introduction

To halt pathogen invasion, plants—unlike animals—solely depend on an innate immune system. They possess an expanded arsenal of cell surface-localized pattern recognition receptors (PRRs) to perceive microbe- and damage-associated molecular patterns (M/DAMPs), here collectively termed invasions patterns [1]. Prominent examples are flagellin sensing 2 (FLS2) and elongation factor Tu (EF-Tu) receptor (EFR), 2 receptor-like kinases (RLKs) with leucine-rich repeat (LRR) ectodomains that initiate defense upon recognition of bacterial flagellin and EF-Tu, respectively [2]. Emerging key players in plant immunity are the lectin receptor kinases, RLKs that are subdivided in 3 distinct classes based on their extracellular lectin domains, i.e., G- (GNA-related or S-locus), C- (calcium-dependent), and L- (legume) type [3]. All 3 are omnipresent in plants but absent in animals, which deploy distinct C-type lectin receptors (CLRs) to initiate innate immunity [4]. Recent years have witnessed an accelerated interest in plant lectin receptors kinases. In this Pearl, we summarize our current knowledge on L-type lectin receptor kinases (LecRKs) in plant immunity.

## Signatures of adaptive evolution

LecRKs are widespread in vascular plants, but only a few have been identified in lower land plants. It remains unclear whether green algae contain genuine LecRKs, because the few listed previously all lack an extracellular lectin domain [5]. The current wealth of plant genomic data enables identification of LecRKs on a genome-wide scale [6–10]. Phylogenetic analyses of LecRKs in 5 Brassicaceae species showed that all contain the 9 distinct clades (I to IX) as initially delineated in *Arabidopsis thaliana* [3,11]. In contrast, clade III orthologs are absent in closely related sister species, whereas outgroup species even lack multiple clades [11]. Clades I, II, and III are thus specific to Brassicaceae and potentially harbor genes with lineage-specific traits. Evolutionary analysis revealed that gene expansion within these lineage-specific clades is driven by a complex interplay of polyploidy and local duplication events [11]. In comparison to the genome-wide average, *Arabidopsis* LecRKs are significantly enriched in duplicates, with an overrepresentation of tandem duplicates, of which most share whole-genome and gene-transposed duplication events. Moreover, LecRKs that evolved by tandem duplication show accelerated molecular evolution, suggesting strong diversifying or positive selection [11]. Similar dynamic evolution is not found in the nucleotide-binding leucine-rich repeat (NLR) resistance gene family or in a few other gene families analyzed in this manner. How this compares to other RLK gene families is as yet unknown.

**Fig 1:**
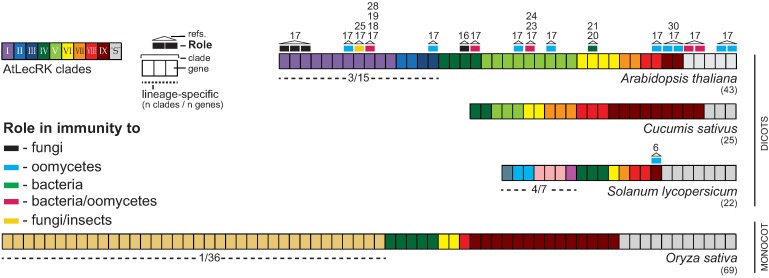
Clade composition of LecRK families in 4 different plant species. Lineage-specific clades are indicated with dashed lines. LecRKs involved in immunity are marked with colored pins. Numbers between brackets represent the amount of LecRKs with typical domain features, i.e., an extracellular lectin domain and an intracellular kinase domain. “S” represents singletons, LecRKs that do not belong to clades I–IX and group distantly.

Comparative analysis revealed that distant species also differ in clade composition. In comparison to cucumber, tomato lacks clade V homologs but has 7 more divergent LecRKs divided over 4 clades (Fig 1) [6,7]. These 4 are also found in *Nicotiana benthamiana* and are likely Solanaceae-specific [6]. In the more distant monocots, the clade composition is again different [8,9]. For example, rice has 4 clades in common with *Arabidopsis* but has a large species-specific clade of 36 LecRKs that largely expanded by tandem duplication [9]. It can thus be concluded that the LecRK superfamily underwent multiple lineage- and species-specific expansions, indicative of genetic adaptation to environmental factors during the course of evolution.

## LecRKs in plant immunity

*LecRKs* are differentially expressed during plant growth and upon exposure to external stimuli, pointing to a wide array of functions. To date, few LecRKs are associated with functions in plant development or abiotic stress tolerance [12–16], and no altered morphology or abiotic stress sensitivity was found in a large set of *Arabidopsis* mutants representing 36 out of the 45 LecRKs [17]. Multiple studies, however, revealed roles in plant immunity (Fig 1), with the first one addressing *Arabidopsis* LecRK-I.9 published in 2011 [18]. Mutants deficient in *LecRK-I*.*9* were found to be compromised in cell wall-associated defense and jasmonate signaling and showed increased susceptibility to the bacterium *Pseudomonas syringae* and the oomycete pathogens *Phytophthora brassicae* and *Phytophthora capsici* [18,19]. Accordingly, overexpression of *LecRK-I*.*9* enhanced resistance to these 3 pathogens. *Arabidopsis* LecRK-VI.2 and LecRK-V.5 were both found to function in stomatal immunity against bacterial pathogens, albeit via different mechanisms. *LecRK-VI*.*2* mutants are unable to inhibit bacterial invasion via stomatal closure and are therefore more susceptible to bacterial pathogens but not to fungi or oomycetes [17,20,21]. This LecRK was found to associate with the flagellin receptor FLS2, emphasizing its specificity in bacterial immunity [22]. Instead of open stomata, *LecRK-V*.*5* mutants have closed stomata. This is promoted by elevated levels of oxidative burst, and as a result, these mutants are resistant to bacterial pathogens [23,24]. In contrast, *LecRK-V*.*5* mutants are more susceptible to *Phytophthora* [17], suggesting a dual role for LecRK-V.5 in defense to different pathogens. As yet, the molecular mechanisms that underlie this Janus-faced role in immunity are unknown. Other immunity-related *Arabidopsis* LecRKs were pinpointed by exposing LecRK mutants to different pathogens. Fourteen *Arabidopsis* LecRKs function in *Phytophthora* resistance, 5 of which also play roles in immunity to bacterial pathogens [17–24]. Five others are implicated in fungal resistance [16,17], including 1 that functions in the perception of insect-egg deposition [25]. Homologs of clade IX LecRKs in tomato and *N*. *benthamiana* also function as immune receptors against *Phytophthora* [6], whereas a LecRK in pepper positively regulates immunity to diverse plant pathogens [26]. These data indicate that LecRKs have a conserved role in immunity throughout the plant kingdom.

## Sensing invasion

The extracellular lectin domains of LecRKs have poorly conserved carbohydrate-binding residues but do compose a conserved hydrophobic cavity that is proposed to recognize hydrophobic ligands, e.g., complex glycans, hormones, and microbial invasion patterns [3]. Whether or not they bind monosaccharides or have agglutination activity similar to soluble legume lectins is unknown. So far, only ligands for 1 LecRK have been reported. *Arabidopsis* LecRK-I.9 was initially identified as a host target of the *Phytophthora infestans* effector IPI-O that interacts with LecRK-I.9 via the tripeptide Arg-Gly-Asp (RGD), a well-known cell attachment motif in mammalian cells [27]. Binding to IPI-O depends on 2 RGD-binding heptamers in the lectin domain [27]. The RGD-binding activity suggested a role for LecRK-I.9 in the maintenance of cell wall–plasma membrane (CW-PM) adhesions. Indeed, loss of LecRK-I.9 disrupts CW-PM integrity and host defense. This behavior as well as impaired resistance to *P*. *syringae* and *Phytophthora* pathogens was found to be phenocopied in *ipiO*-expressing *Arabidopsis* [18].

A second ligand is extracellular ATP (eATP). *LecRK-I*.*9* mutants are deficient in response to ATP and hence named “does not respond to nucleotides 1” (dorn1) [28]. In in vitro binding assays, the ectodomain of LecRK-I.9 has a relatively high binding affinity for ATP [28]. Mammalian purinoceptors that sense eATP were discovered many years ago and are well studied. Plants, however, have no homologs of purinoceptors. LecRK-I.9 is thereby the first identified eATP receptor in plants. Plants release eATP upon pathogen attack, and hence a model was proposed in which eATP acts as an invasion pattern that is recognized by LecRK-I.9 [28,29].

## Launching defense

Our knowledge on immune signaling complexes is largely based on studies on the paradigmatic LRR-RLKs FLS2 and EFR [2]. Both heterodimerize with BRI1-associated receptor kinase 1 (BAK1)—a central coreceptor in defense signaling—in a ligand-dependent manner, and this leads to a cascade of phosphorylation events ultimately culminating in immunity. How do LecRKs activate defense and what are their downstream targets? Thus far, 2 LecRK interactors have been identified. One of them is ABCG40; a G-family ATP-binding cassette (ABC) transporter implicated in transport of the phytohormone abscisic acid (ABA). It was found to interact with *Arabidopsis* clade IX LecRKs that function in *Phytophthora* resistance and is required to mount adequate resistance [30]. Also, other LecRKs have links with ABA. LecRK-V.5 negatively regulates ABA-mediated stomatal closure, whereas LecRK-V.1 and clade VI LecRKs impact ABA responses during seed germination [14,15]. The second one is FLS2, which interacts with LecRK-VI.2 in a ligand-independent manner. It is remarkable, however, that loss of *LecRK-VI*.*2* does not affect FLS2-mediated association with BAK1 or phosphorylation of the receptor-like cytoplasmic kinase, botrytis-induced kinase 1 (BIK1) [22]. Since BIK1 is activated upon ligand binding, LecRK-VI.2 seems to function independently or downstream of BIK1. Interestingly, BAK1 is dispensable for *Arabidopsis* immunity mediated by lipooligosaccharide-specific reduced elicitation (LORE), a G-type LecRK that perceives bacterial lipopolysaccharides [31]. Whether LecRKs require coreceptors to mediate immunity remains to be determined.

Receptor dimerization is often a critical step in defense signaling [2]. As such, homo- or heterodimerization of LecRKs can also be hypothesized as well as the option that their lectin domains dimerize with legume lectin-like proteins (LLPs). *Arabidopsis* LLPs are most homologous to clade VII and VIII LecRKs and also lack the essential residues for monosaccharide binding [11]. A role of these apoplastic LLPs in plant immunity is supported by the finding that overexpression of SA-induced legume lectin-like protein 1 (SAI-LLP1/LecP-I.7) in *Arabidopsis* potentiates defense to *P*. *syringae* [32]. Complex formation based on dimerization of extracellular domains has been observed for chitin elicitor receptor kinase 1 (CERK1), a lysin motif (LysM)-type lectin receptor kinase that perceives fungal chitin oligomers as an invasion pattern [2]. Upon chitin binding, *Arabidopsis* CERK1 forms homodimers, which activates intracellular signalling leading to fungal resistance. CERK1 also forms chitin-induced heterodimers with LYK5, an *Arabidopsis* LysM receptor kinase with an even stronger chitin-binding affinity. Interestingly, CERK1 also mediates immunity triggered by bacterial peptidoglycan [2]. CERK1 homodimers in rice, however, are unable to bind chitin directly and require heterodimerization with the chitin elicitor-binding protein (CEBiP), a membrane-spanning LysM protein that also lacks a kinase domain [2]. Another notable example is S-locus receptor kinase (SRK), a G-type LecRK that determines self-incompatibility in Brassicaceae by recognizing the pollen ligand S-locus cysteine-rich protein (SCR) during incompatible pollination [33]. Unlike CERK1, SRK can exist *in planta* as a homodimer prior to ligand binding. Structural studies recently revealed that SRC binding enhances homodimerization of SRK [34]. SRK also associates with S-locus glycoprotein (SLG), an apoplastic protein with a highly similar lectin domain, but whether SLG participates in a SCR-binding complex is still unknown [33]. In this respect, the multitude of LecRKs and LLPs (45 and 11 in *Arabidopsis*, respectively) may offer a wealth of combinatorial possibilities of receptor heterodimerization.

## Exploiting LecRKs for crop resistance

LecRKs have several features that make them attractive as potential resistance components. They form a vast and diverse gene family, several of which confer broad-spectrum disease resistance. Another advantage is that LecRKs retain their function in plant immunity after interfamily transfer in a similar way as demonstrated for the LRR-RLK EFR [35]. This was shown for 3 *Arabidopsis* LecRKs when ectopically expressed in solanaceous plants [22,36,37]. As such, *LecRK*s have the potential to be introduced into distantly related species to increase disease resistance. A disadvantage, however, is that LecRK-mediated resistance is relatively mild in comparison to strain-specific resistance governed by intracellular NLR resistance proteins. Resistance can be enhanced by boosting *LecRK* expression, but caveats are pleiotropic effects on plant fitness [36,37]. For instance, ectopic expression of *LecRK-IX*.*1* can induce cell death. Fortunately, cell death in this case could be uncoupled from its resistance function [30,37], and this opens possibilities to minimize cell death without losing LecRK-mediated resistance. Exploiting LecRKs to improve crop resistance is within reach, especially when taking advantage of high throughput screening of TILLING populations or gene-editing tools such as CRISPR/Cas9. LecRKs can be used as a novel resource of cell surface receptors to boost immunity, and pyramiding LecRKs with classical intracellular NLRs may lead to a broader and more durable disease resistance.

## Open questions

Our current understanding of how LecRKs function in plant defense is in its infancy, with many questions unanswered. Because LecRKs are largely diverse, they might recognize a variety of invasion patterns. Can the thus far detected immunogenic ligands be extrapolated to other LecRKs? Are there ligands that are specific for the lineage-specific LecRKs? Are other *Arabidopsis* LecRKs, besides LecRK-I.9, sensing eATP and RGD? And what is the eATP receptor in plants that lack clade I LecRKs? The fact that LecRK-I.9 binds both RGD and eATP brings into question how these 2 processes are regulated. In this respect, it is of note that the mammalian eATP receptor P2Y_2_ functions through interaction via its RGD motif with integrins that mediate cell integrity [38]. What type of components interact with the kinase domain of LecRKs? It remains elusive which components reside within LecRK signaling complexes. What is the role, if any, of common signaling partners, such as BAK1 and BIK1, and what is phosphorylated by LecRKs? It is also important to understand how microbial pathogens deal with LecRK-mediated immunity. Can pathogens compete via effectors for ligand binding to interfere with LecRK-mediated defense, or do they employ effectors that target LecRKs for degradation to promote host colonization? It is envisioned that integration of genetic, biochemical, and phylogenomics approaches will allow us to answer the abovementioned questions and support future resistance breeding.
